# La aplicación de las ómicas para comprender la base molecular de la insuficiencia hepática aguda sobre crónica

**DOI:** 10.1515/almed-2021-0068

**Published:** 2021-11-11

**Authors:** Joan Clària

**Affiliations:** Servicio de Bioquímica y Genética Molecular, Hospital Clínico de Barcelona, C/ Villarroel 170, 08036, Barcelona, España

**Keywords:** enfermedad hepática avanzada, inflamación sistémica, inmunometabolismo, inmunosupresión, mediadores lípidicos bioactivos

## Abstract

La insuficiencia hepática aguda sobre crónica (ACLF) es un síndrome complejo que desarrollan los pacientes con cirrosis aguda descompensada. En esta patología, un sistema inmune desequilibrado y la excesiva inflamación sistémica están estrechamente relacionados con el fallo orgánico y la mortalidad a corto plazo. En la presente revisión, describimos la contribución de las llamadas tecnologías “ómicas” a la caracterización del estado hiperinflamatorio en pacientes con cirrosis descompensada aguda que han desarrollado ACLF, centrándonos en el papel de la metabolómica, la lipidómica y la transcriptómica en la identificación de los factores desencadenantes (patógenos y patrones moleculares asociados al daño [PAMPs y DAMPs]), así como de las moléculas efectoras (citocinas, quimiocinas, factores de crecimiento y mediadores lipídicos bioactivos) que provocan la activación del sistema inmune innato. Esta revisión también describe el papel esencial que pueden desempeñar las ciencias “ómicas” a la hora de acelerar la identificación de nuevos biomarcadores, que podrían dar lugar a la implementación de nuevas terapias o intervenciones destinadas a proteger a estos pacientes de la excesiva inflamación sistémica, así como del fallo orgánico.

## Insuficiencia hepática aguda sobre crónica (ACLF)

La ACLF es un síndrome grave al que se asocia una elevada mortalidad a corto plazo que desarrollan los pacientes con cirrosis hepática con descompensación aguda (DA). La ACLF se caracteriza por la presencia de disfunción orgánica e insuficiencia hepática, renal, cerebral, y de los sistemas respiratorio, circulatorio y de coagulación, definidos conforme a los criterios de la Asociación Europea para el Estudio del Hígado y el Consorcio sobre la Insuficiencia Hepática Crónica (EASL‐CLIF) [1], [2], [3]. La ACLF se clasifica en tres grados de gravedad (ACLF-1, -2 y -3) dependiendo del número de fallos orgánicos. Tal como establecen los criterios EASL‐CLIF, en la ACLF de grado 1 se incluyen los pacientes con insuficiencia renal, hepática, circulatoria o respiratoria, o coagulopatía asociada a niveles de creatinina de entre 1,5 y 1,9 mg/dL o encefalopatía hepática de grado I o grado II o ambos, así como los pacientes con insuficiencia cerebral con niveles de creatinina de entre 1,5 mg/dL y 1,9 mg/dL. La ACLF de grado 2 se establece con la presencia de dos fallos orgánicos En la ACLF de grado 3 se incluyen los pacientes con tres o más fallos orgánicos. El estudio CANONIC, un estudio observacional prospectivo en 1343 pacientes hospitalizados por descompensación aguda de la cirrosis, definida según los criterios basados en la evidencia de ACLF, que incluyen la presencia de uno o varios fallos orgánicos y un riesgo de mortalidad a los 28 días del 15% o más [1]. En los países occidentales, la ACLF tiene especial prevalencia entre los pacientes jóvenes con enfermedad hepática alcohólica y, en el 60% de los casos, está causada por infecciones bacterianas o alcoholismo activo. En los países asiáticos, la ACLF se suele diagnosticar en pacientes con cirrosis relacionada con la hepatitis B, con una prevalencia más baja de insuficiencia orgánica extrahepática.

## La inflamación sistémica es un signo distintivo de la ACLF

Hallazgos recientes que han permitido conocer mejor la patofisiología de la ACLF demuestran la asociación entre la inflamación sistémica y el daño orgánico en pacientes con cirrosis AD que desarrollan ACLF [4], [5], [6]. Este estado hiperinflamatorio sistémico lo causa la producción en masa de mediadores inflamatorios como las citocinas, las quimiocinas, los factores de crecimiento y los mediadores lipídicos bioactivos, que tienen capacidad para inducir daño tisular mediado por la respuesta inmune, un proceso llamado inmunopatología. Por ejemplo, en la microvasculatura de los órganos vitales, las citocinas proinflamatorias dañan el glucocáliz endotelial, provocando la adhesión de los neutrófilos y los monocitos a las células endoteliales y su transmigración a los tejidos [7]. Las células inmunes activadas, a su vez, liberan mediadores como proteasas, moléculas oxidativas, citocinas citotóxicas y mediadores lipídicos de inflamación (esto es, prostaglandinas (PG) y leucotrienos (LT)) que intensifican el daño tisular.

**Figura 1:**
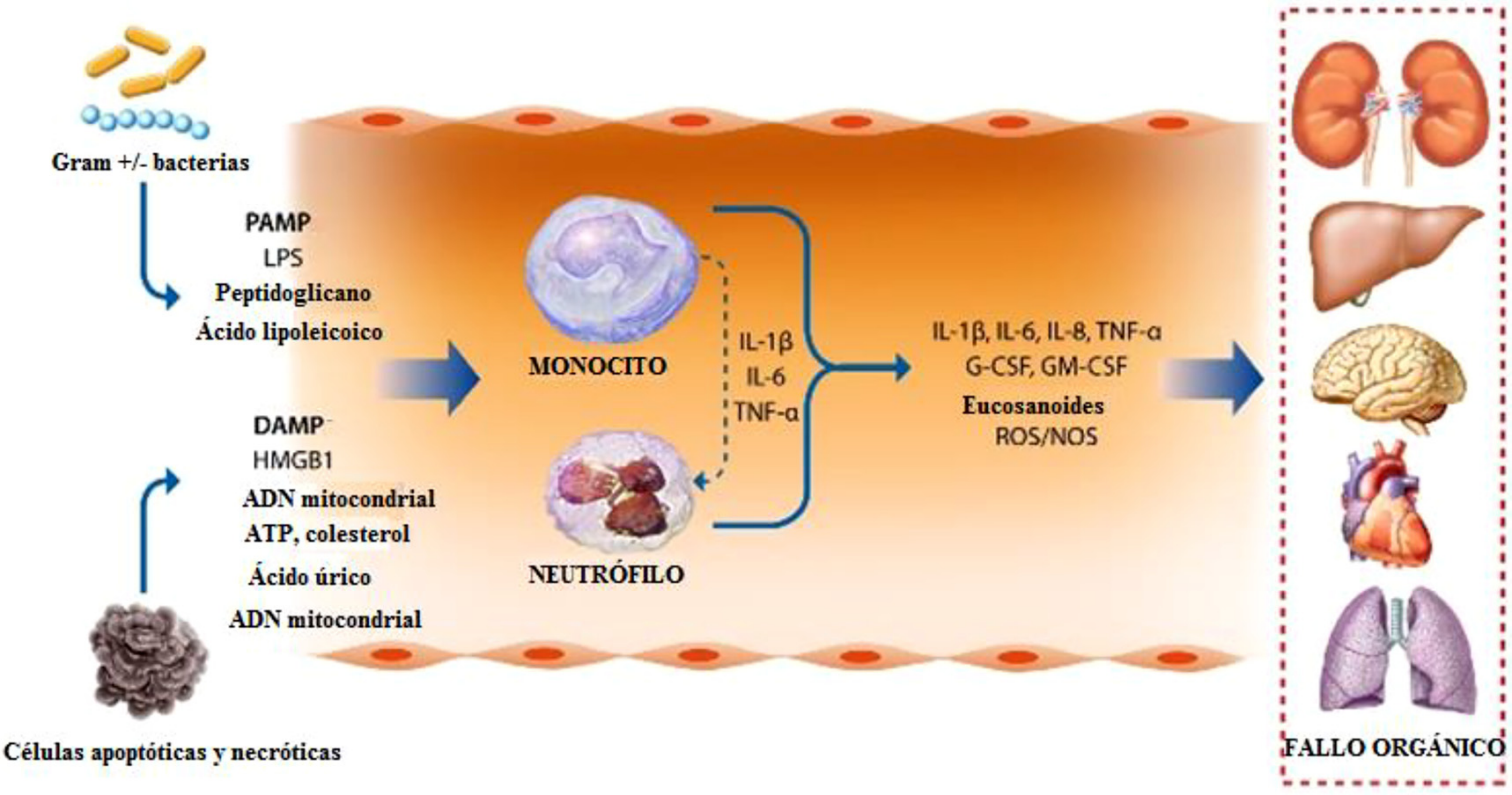
Esquema de la función de los patrones moleculares asociados a patógenos (PAMP) liberados por bacterias Gram+/− (lipopolisacárido (LPS), peptidoglucanos y ácido lipoteicoico) y de los patrones moleculares asociados a daño (DAMP) derivados de células necróticas o apoptóticas moribundas (proteínas de alta movilidad del grupo 1 (HMGB1), fragmentos de ADN mitocondrial, histones, ATP, colesterol y cristales de urato).

Actualmente se desconoce la identidad de los factores desencadenantes (si son de origen infeccioso o no) que provocan una inflamación sistémica excesiva en los pacientes con cirrosis AD evolucionada a ACLF. El 33% de los pacientes con ACLF presentan infecciones bacterianas [8] por lo que los patrones moleculares asociados a patógenos (PAMP) producidos por las bacterias infecciosas probablemente contribuyen a dicha inflamación (Figura 1). Además, los PAMP circulantes podrían ser el resultado de la translocación de productos bacterianos desde la luz intestinal hacia la circulación sistémica. De hecho, los pacientes con cirrosis AD que desarrollan ACLF suelen presentar sobrecrecimiento bacteriano, mayor permeabilidad de la mucosa intestinal y disfunción del sistema inmunológico innato intestinal [9]. Las PAMP, son estructuras moleculares muy conservadas que el organismo anfitrión reconoce a través de unos receptores específicos llamados receptores de reconocimiento de patrones (PRR), entre los que se encuentran, entre otros, los receptores tipo Toll (TLR), presentes en la superficie celular o en el compartimento endosómico, y los receptores tipo NOD, presentes en el citosol de las células inmunes [10]. Estos receptores reconocen los ácidos nucleicos y las proteínas, lípidos y carbohidratos característicos de las bacterias y los virus. La activación de los PRR estimula las cascadas de señalización que activan factores de transcripción como el factor nuclear (NF)-kB o la proteína activadora-1 [11], que, a su vez, inducen la expresión de una batería de genes codificantes de las moléculas implicadas en la inflamación (esto es, la interleucina (IL)-6) y el factor de necrosis tumoral (TNF) α). La inflamación sistémica puede darse en pacientes con cirrosis AD y ACLF, en ausencia de infecciones bacterianas y/o translocación bacteriana, como resultado de la producción de patrones moleculares asociados al daño (DAMPs) por parte de los órganos y tejidos dañados (Figura 1). Los DAMP los producen las células muertas, moribundas o dañadas, y se originan en varios compartimentos celulares, especialmente en el núcleo (proteínas de alta movilidad del grupo 1 (HMGB1) e histones), los mitocondrios (ADN mitocondriales y los péptidos de formilo) y el citosol (adenosín trifosfato (ATP)) [11].

La respuesta hiperinflamatoria sistémica en los pacientes con ACLF se suele ser concomitante a un sistema inmunológico innato disfuncional a nivel humoral, físico y celular [6, 12, 13]. Debido a la insuficiencia hepatocelular, los pacientes con cirrosis suelen presentar una capacidad contra la inmunidad humoral reducida, como resultado de una menor producción de proteínas de fase aguda, hipoalbuminemia y un sistema del complemento defectuoso [14, 15]. Así mismo, en la cirrosis AD, y más aún en la ACLF, se ve afectada la barrera física, siendo la translocación bacteriana y la disfunción de la vasculatura y el endotelio sinusoidal sus características más frecuentes [6]. Teniendo en cuenta todos estos componentes del sistema inmunológico innato, el estado inmunológico general de los pacientes con ACLF puede variar entre un estado inmunosupresor/inmunorregulador/tolerogénico y un estado fuertemente hiperinflamatorio, extremos que no son excluyentes entre sí.

## Mediadores solubles de inflamación en la ACLF

Tal como se ha descrito anteriormente, el sistema inmunológico innato incompetente en pacientes con ACLF se caracteriza por mayores niveles circulantes de proteínas pequeñas (citocinas, quimiocinas y factores de crecimiento) y pequeños lípidos bioactivos (mediadores de lípidos), que activan respuestas inflamatorias e inmunológicas descontroladas.

### Análisis multiplex de citocinas, quimiocinas y factores de crecimiento

Las citocinas son glicoproteínas de bajo peso molecular que orquestan la efectividad de la inmunidad innata induciendo inflamación local y respuestas sistémicas agudas [16, 17]. La producción de citocinas por parte de los leucocitos es uno de los primeros pasos de la cascada inflamatoria. Una vez liberadas, las citocinas se unen a receptores específicos en sus células diana [18]. Aunque los leucocitos son la principal fuente de producción de citocinas, existe cada vez más evidencia científica sobre el papel de las células parenquimales en la producción de citocinas inflamatorias, interactuando con los leucocitos para optimizar las respuestas inmunes [19]. Las citocinas también son importantes a la hora de iniciar, amplificar y mediar la inmunidad adaptativa [20]. Las citocinas se pueden clasificar en diferentes familias, como la familia de los TNF, las IL-6 y la familia de interferones (IFN) α, β, e γ. Las citocinas también se pueden clasificar según su papel como mediadores proinflamatorios o anti-inflamatorios. Los TNF-α, IL-1β e IL-6 son citocinas proinflamatorias bien caracterizadas, mientras que los antagonistas de los receptores (IL-1ra) de IL-4, IL-10 e IL-1 se consideran anti-inflamatorioss [20]. La presencia de mayores niveles circulantes de TNFα e IL-6 en pacientes con cirrosis sin infecciones se describió hace décadas [21, 22], aunque no ha sido hasta hace poco cuando se ha realizado una caracterización más completa de estos mediadores inflamatorios en la ACLF y su correlación con la gravedad de la enfermedad y los fallos orgánicos [4, 5]. En este contexto, una de las principales citocinas es la IL-6, que es una citocina pleiotrópica producida en respuesta a las infecciones y al daño tisular. El lipopolisacárido (LPS), la IL-1β o el TNF-α estimulan la producción de la proteína TLR4, que a su vez induce la síntesis y secreción de IL-6, uno de los principales factores que estimulan la liberación de proteínas hepáticas de fase aguda. La IL-6 está fuertemente correlacionada con el desarrollo de disfunción renal y mortalidad en pacientes con cirrosis y peritonitis bacteriana [23]. Un aspecto importante a tener en cuenta es que en pacientes con ACLF, los niveles circulantes no solo de IL-6, sino de la mayoría de las citocinas, son de la misma magnitud que los descritos en pacientes con sepsis. Por lo tanto, el término “tormenta de citocinas”, que hace referencia a la producción desmesurada de estos mediadores como consecuencia de la sobreactivación del sistema inmune acompañada de inflamación sistémica, frecuente en las patologías sépticas [16], también es pertinente en la ACLF.

### Análisis de los niveles circulantes de pequeños mediadores lipídicos bioactivos mediante la lipidómica dirigida

Los mediadores lipídicos bioactivos provienen de especies de lípidos estructurales (fosfolípidos que contienen ácidos grasos poliinsaturados [AGPI]), que componen la bicapa lipídica de las membranas celulares [24]. La mayoría de los mediadores lipídicos se derivan de los AGPI omega-3 y omega-6, que son liberados a demanda por la fosfolipasa A_2_ al citosol, en respuesta al estímulo inflamatorio de la membrana celular [24], [25], [26]. En el citosol, las vías enzimáticas de ciclooxigenasa (COX), lipoxigenasa (LOX) y citocromo P450 (CYP) convierten los AGPI en diversos mediadores lipídicos biológicamente activos, que se liberan para que actúen como autacoides [24], [25], [26]. El ácido araquidónico (AA), un ácido graso esencial poliinsaturado (omega-6), es el sustrato para la biosíntesis intracelular de una de las principales familias de mediadores lipídicos: los eicosanoides. La familia de los eicosanoides se compone de PG, tromboxano A2 (TXA2), LT, lipoxinas (LXs) y ácidos epoxieicosatrienoicos (EETs). Con la excepción de las LX, los eicosanoides poseen propiedades proinflamatorias y, de hecho, los PG y el TXA_2_ son las principales dianas de los fármacos antiinflamatorios no esteroides (AINE) [27, 28]. Al igual que las citocinas, los eicosanoides son liberados en grandes cantidades por los leucocitos, en respuesta a infecciones o al daño tisular provocados por la llamada “tormenta eicosanoide” [24].

Los eicosanoides y, concretamente, la prostaglandina E₂ (PGE_2_) desempeñan un papel fundamental en el desarrollo de los cinco signos cardinales de inflamación: edema, eritema, dolor, fiebre e impotencia funcional. La PGE_2_ incrementa la permeabilidad vascular, contribuyendo a la extravasación de fluidos y a la aparición de edema (inflamación) [29]. Además, la PGE_2_ sensibiliza las terminaciones de los nervios sensoriales periféricos situados en el lugar de la inflamación, actuando en la médula espinal para evocar hiperalgesia y dolor [29]. La PGE_2_ también es crucial para la aparición de fiebre, siendo la piresis la consecuencia de los niveles elevados de PGE_2_ en el sistema nervioso central, originados por las acciones de la IL-1α producida por las células inmunes activadas en la circulación sistémica [30]. Finalmente, la PGE_2_ ejerce amplias funciones inmunosupresoras dependiendo de su lugar de acción y formación, siendo un mediador clave en la disfunción de las células mieloides, que inhibe la eliminación de bacterias por la nicotinamida adenina dinucleótido fosfato (NADPH) oxidasa y la fagocitosis mediada por FcγR [31, 32]. Por otro lado, los LT están complejamente implicados en las reacciones alérgicas e inflamatorias, siendo potentes mediadores de la inflamación. Por ejemplo, el LTB_4_ induce la adhesión endotelial, la quimiotaxis y la activación de los leucocitos, la secreción de enzimas lisosomales y la producción de anión superóxido en los neutrófilos [33, 34]. Además, existen evidencia de que el LTB_4_ estimula la síntesis de citocinas [34]. Los LTC_4_/LTD_4_/LTE_4_ son potentes quimioatrayentes de eosinófilos, causan la fuga de plasma de las vénulas postcapilares, mejoran la secreción de mucosidad e inducen la síntesis y liberación de mediadores proinflamatorios, entre los que se incluyen la IL-8 y el factor de activación plaquetaria [26, 34].

Al igual que el AA (AGPI omega-6), algunos ácidos grasos poliinsaturados omega-3, como el ácido eicosapentaenoico (AE) y el ácido docosahexaenoico (AD), también son convertidos en mediadores de lípidos bioactivos por las vías COX, LOX y CYP aunque, en este caso, ejercen una potente acción anti-inflamatoria y pro-resolutiva [35], [36], [37], [38]. Estos mediadores, que suelen recibir el nombre de “mediadores pro-resolutivos especializados” (SPM, por sus siglas en inglés), recientemente han atraído la atención de la comunidad científica, ya que no solo actúan como “señales de frenado” de inflamación persistente, sino que también desempeñan una importante función en la resolución dinámica de la inflamación de los tejidos [35], [36], [37], [38]. Concretamente, las enzimas COS y LOX pueden generar uno mediadores SPM llamados resolvinas, un producto de la interacción entre células inmunes durante la fase de resolución de la inflamación. Estas se clasifican en resolvinas de la serie D, si son derivadas del DHA, o resolvinas de la serie E si la biosíntesis se inicia en el EPA [35, 36]. Por otro lado, las enzimas LOX pueden convertir el DHA en otro tipo de mediadores SPM llamados protectinas (PD1) y maresinas (MaR1 y MaR2) [35, 36]. Finalmente, el ácido docosapentaenoico (DPA) (AGPI omega-3) también se puede metabolizar a resolvinas de la serie 13 (RvTs) [39].

Los mediadores SPM ejercen una doble función como señales de detención de la inflamación y como activadores de la resolución de la inflamación [35, 36]. Cada mediador SPM tiene unas propiedades antiinflamatorias específicas. Por ejemplo, la RvE1 reduce la infiltración de neutrófilos y la migración de células T, reduce la secreción de TNFα e IFNα, inhibe la formación de quimiocina y bloquea la activación de NF-kB mediada por IL-1 β [40]. RvD1 y RvD2 reducen el dolor inflamatorio, bloquean la expresión de IL-1β inducida por TNFα y limitan la infiltración de leucocitos polimorfonucleares en el cerebro, piel y peritoneo inflamados [41, 42]. Concretamente, RvD2 ha demostrado ser un potente regulador endógeno de las respuestas inflamatorias excesivas en ratones con sepsis microbiana [43]. Además, RvD2 reduce la expresión de IL-1β, la formación de multímeros de ASC (que median la agregación de los inflamasomas) y la secreción de IL-1β maduras en los macrófagos producidos en el peritoneo y la médula espinal [44]. Por otro lado, PD1 y MaR1 ejercen acciones protectoras en modelos agudos de inflamación bloqueando la migración e infiltración de PMN en el lugar de la inflamación [35, 45]. Además de sus propiedades antiinflamatorias, los SPM ejercen potentes acciones proresolutivas y aceleran el proceso de resolución también en rango nanomolar. En general, los SPM allanan el camino para la diferenciación de los monocitos en macrófagos fagocíticos, facilitando la eliminación de las células muertas o moribundas, así como la depuración de bacterias [46]. Por ejemplo, RvE1 estimula la fagocitosis de PMN apoptóticos por macrófagos y es un potente contrarregulador de la expresión de L-selectina [40, 47]. Cabe señalar que la fagocitosis de células apoptóticas por macrófagos también induce la biosíntesis de SPM, que actúan de forma autocrina para facilitar la fagocitosis [48].

Hasta hace poco se desconocía el papel de los mediadores lipídicos en la ACLF. Algunos estudios muestran que los niveles plasmáticos de ácidos grasos están aumentados en pacientes con cirrosis AD y ACLF [49], aunque entre el repertorio de ácidos grasos, los ácidos grasos poliinsaturados se ven invariablemente reducidos en pacientes con ACLF [50]. Esta supresión se puede observar al medir el contenido total plasmático, que representa con mayor precisión el perfil de AGPI en la circulación y no se aprecia cuando solo se analizan los AGPI libres circulantes [51]. Además, los pacientes con ACLF muestran un notable desequilibrio entre las familias de AGPI omega-6 y omega-3 (mayor proporción de AA (omega-6) con respecto a EPA (omega-3)), que es un marcador subrogado de inflamación sistémica y/o resolución alterada [51]. En relación con la resolución alterada, a finales de los años noventa, nuestro grupo describió un déficit en la formación de LXA_4_ en coincubaciones de PMN y plaquetas en pacientes con cirrosis AD, frente a sujetos sanos [52]. Además, en los leucocitos PMN de estos pacientes, detectamos en una respuesta quimiotáctica defectuosa frente a LTB_4_, una anomalía que probablemente contribuye a la disfunción bactericida característica de esta patología [52]. O’Brien y col demostraron que PGE_2_ ejerce un papel fundamental en la inducción del estado inmunosupresor de los pacientes con cirrosis AD y ACLF, que expone a estos pacientes a un mayor riesgo de infecciones recurrentes [53]. En un estudio de lipidómica dirigida realizado en 49 pacientes del estudio ATTIRE, el mismo grupo de investigadores también exploró el potencial de las infusiones de albúmina sérica a la hora de restaurar la función inmune en pacientes con cirrosis AD y ACLF, los efectos de la albúmina en la unión de PGE_2_ y su potencial interacción con los mediadores lipídicos pro-resolutivos [54]. El hallazgo más sorprendente de este estudio fue que se pueden distinguir dos fenotipos de pacientes con cirrosis AD según su perfil de mediadores lipídicos. Un grupo mostró un perfil hipoactivado con concentraciones reducidas de varios mediadores pro-resolutivos y pro-inflamatorios, entre los que se encuentran PD1, LXA_4_, PGs, TXB_2_ y LTB_4_, mientras que los otros mostraron un estado hiperactivado con mayor producción de estos mediadores lipídicos [54]. El segundo grupo mostró un elevado recuento de hematíes, una mayor temperatura y niveles aumentados de proteína C-reactiva (PCR) y citocinas, acompañados de mayores concentraciones de LPS plasmático. Estos investigadores también identificaron una tendencia distinta en las vías que inician la inflamación y las vías que la resuelven, entre los pacientes con cirrosis AD supervivientes y los no supervivientes, lo que indica que la supervivencia también se asocia al perfil alterado de niveles de SPM [55]. Son necesarios más estudios para determinar el significado patofisiológico de estos hallazgos y si pueden resultar útiles a la hora de monitorizar la respuesta a terapia en la práctica clínica.

**Figura 2:**
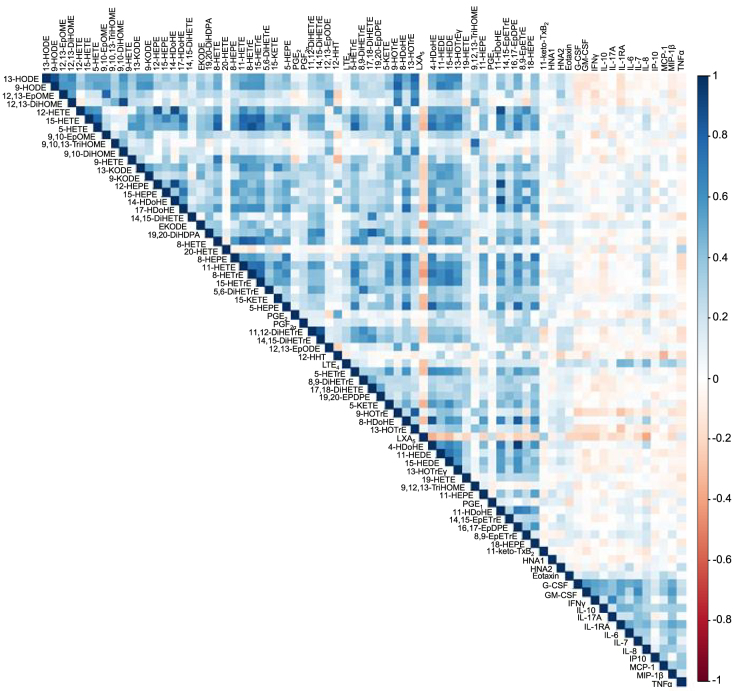
Matriz de correlación entre mediadores lipídicos y marcadores inflamatorios en pacientes con cirrosis aguda descompensada. Datos extraídos de la referencia [50].

A la luz de la magnitud y diversidad de la red de mediadores lipídicos, una caracterización adecuada de dichas moléculas en patologías complejas como la ACLF requiere la aplicación de las ómicas en estudios con una amplia muestra de pacientes como la cohorte CANONIC. Recientemente realizamos una aproximación lipidómica aplicando la cromatografía líquida acoplada a la espectrometría de masas (LC-MS/MS) para cribar 100 mediadores lipídicos en 246 pacientes con cirrosis AD, 119 de los cuales procedían del estudio CANONIC y padecían ACLF [50]. En total se detectaron 59 mediadores lipídicos (de 100) en el plasma de los pacientes cirróticos. Un análisis integrado reveló la relación entre la ACLF y niveles circulantes elevados de las familias de LT, PG, ácidos grasos epoxi- ceto y TX, concurrentes a niveles reducidos de LX y ácidos grasos epoxi. De los 59 mediadores lipídicos anotados, 16 mostraron una asociación significativa con la gravedad de la enfermedad. De estos, 11 mediadores lipídicos distinguieron a los pacientes de todas las fases (con o sin ACLF) de los sujetos sanos, mientras que dos mediadores lipídicos, LTE_4_ y ácido 12-hidroxiheptadecatrienoico (12-HHT), ambos derivados del AA, dejaron una huella plasmática mínima que discriminaba entre pacientes con y sin ACLF. Además, LTE_4_ fue uno de los principales mediadores lipídicos con regulación diferenciada y niveles gradualmente aumentados en AD y ACLF, con respecto a los sujetos sanos, así como en ACLF-3 frente a ACLF-1 y -2. Curiosamente, los niveles plasmáticos de LTE_4_ aumentaban con la progresión de la enfermedad y disminuían cuando esta mejoraba. Así mismo, se observó una correlación significativa entre LTE_4_ y la quimiocina inflamatoria IL-8 (Figura 2) y el marcador de necrosis/apóptosis queratina 18 (K18). Por otro lado, actualmente se desconoce el papel biológico del ácido 12-HHT, biosintetizado por la sintasa TXA_2_ a una proporción equimolar con respecto a TXA_2_. Anteriormente se consideraba que 12-HHT era un mero bioproducto de la biosíntesis del potente vasoconstrictor TXA_2_, aunque estudios recientes indican que este mediador lipídico puede inducir la quimiotaxis de las células inmunes al unirse al receptor 2 de LTB_4_ [56]. Nuestro estudio de lipidómica dirigida también reveló niveles elevados del eicosanoide proinflamatorio y vasoconstrictor PGF_2α_ en pacientes con ACLF [53]. La PGF_2α_ está ampliamente distribuida en el organismo y es una de las PG más abundantes en las regiones inflamadas, donde ejerce distintas actividades biológicas, incluyendo la contracción de la musculatura lisa bronquial y vascular y la regulación de la secreción de reinina y la presión arterial [57]. Además, la PGF_2α_mostró ser un mediador lipídico con capacidad para distinguir a pacientes con ACLF de sujetos sanos y fue el único mediador lipídico asociado a insuficiencia circulatoria en pacientes con ACLF.

A diferencia de la LTE_4_ y la PGF_2α_, observamos que la LXA_5_ estaba invariablemente reducida en los pacientes con ACLF [50]. LXA_5_ es un SPM derivado del EPA (AGPI omega-3) que promueve la resolución efectiva de la inflamación. La biosíntesis de LXA_5_ por fuentes endógenas de EPA es iniciada por la enzima 15-LOX y principalmente se produce en las células con actividad de 15-LOX, como las del sistema inmune. Dado que los niveles circulantes de LXA_5_ estaban suprimidos en los pacientes con ACLF, mientras que sus niveles de EPA fueron similares a los de los pacientes con cirrosis AD y los sujetos sanos, esto indica un acceso reducido en lugar de limitado a su sustrato biosintético (esto es, EPA). Así mismo, el análisis de expresión génica en los leucocitos confirmó un decremento notable de la expresión de 15-LOX en pacientes con ACLF frente a aquellos con cirrosis AD [50]. Además, los niveles plasmáticos de LXA_5_ mostraron una relación inversamente proporcional a IL-8 (Figura 2) y, junto con el ácido 12,13-epoxi-9-ceto-. 10(trans)-octadecenoico (EKODE) (derivado del ácido lionileico AGPI omega-6), conformaban un perfil asociado al fallo de la coagulación y la insuficiencia hepática en pacientes con ACLF [50]. Otros mediadores lipídicos derivados del ácido linoleico, esto es el ácido 9 (10) -epoxi-9Z-octadecenoico (EpOME) y 12(13)-EpOME, que son indicadores de actividad bactericida efectiva, también estaban significativamente deprimidos en ACLF [50]. En su conjunto, este estudio reveló que la desproporción entre mediadores lipídicos pro-inflamatorios (p.ej. LTE_4_) y mediadores anti-inflamatorios y pro-resolutivos (p.ej. LXA_5_) es un signo característico de inflamación sistémica severa en pacientes con ACLF. Así mismo, este estudio de lipidómica dirigida mostró una asociación entre el patrón de mediadores lipídicos específicos y la gravedad y pronóstico de los pacientes con ACLF.

## Inmunometabolismo e inflamación sistémica en la ACLF

**Figura 3:**
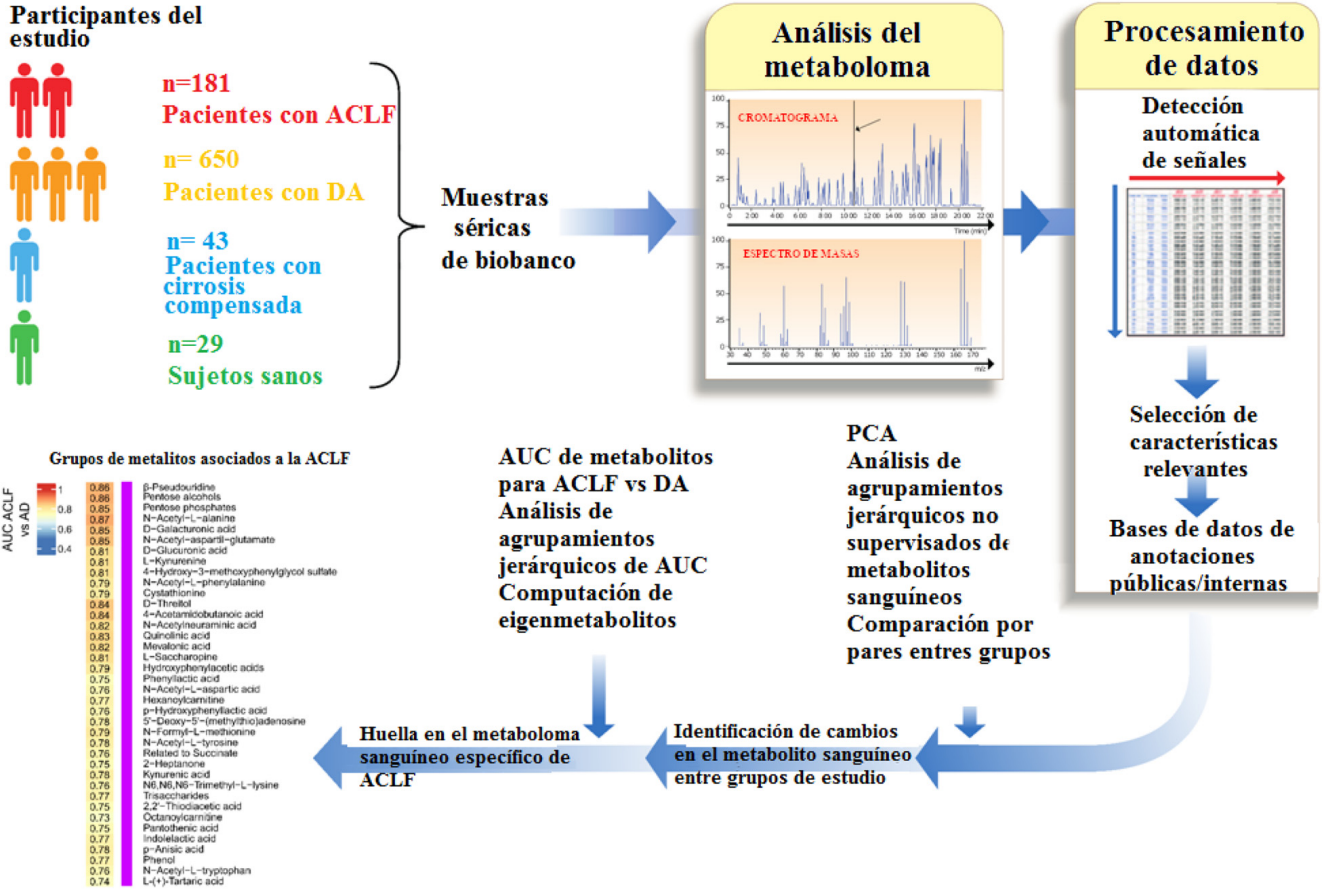
Estrategia empleado en el estudio metabolómico para analizar el metaboloma sanguíneo de pacientes con cirrosis descompensada aguda con y sin ACLF. Los datos de este estudio figuran en la referencia [49].

Las respuestas inflamatorias consumen gran cantidad de energía y requieren la movilización de la reserva de nutrientes para promover la activación inmune, especialmente en un contexto de inflamación sistémica severa como en el caso de la sepsis [58, 59]. La metabolómica, que identifica y cuantifica los metabolitos de bajo peso molecular (el metaboloma), es la ómica más cercana a los fenotipos clínicos [60]. Los metabolitos no solo reflejan la actividad metabólica de los tejidos, sino que, dado que multitud de ellos también desempeñan una potente actividad biológica en procesos patofisiológicos críticos, estos también influyen en el fenotipo clínico. La mejor estrategia a la hora de caracterizar los cambios metabolómicos de síndromes complejos como la ACLF es realizar estudios no dirigidos de alto rendimiento (estudios agnósticos) en amplias series de pacientes. Se han publicado tres estudios recientes en los que se ha aplicado esta estrategia. El primero de ellos es un estudio llevado a cabo por Bajaj y col, que realizaron análisis a 602 pacientes del consorcio NACSELD [61]. El hallazgo más interesante de este estudio fue la identificación de metabolitos de origen microbiano asociados al desarrollo de ACLF. Por su parte, Zacherini y col subrayaron la importancia de los metabolitos derivados de la proteolisis y del catabolismo de los aminoácidos en la ACLF [62]. Finalmente, el tercer estudio, realizado por Moreau y col [49], incluyó más de 831 pacientes reclutados prospectivamente en el estudio CANONIC (Figura 3), cuyos principales resultados son expuestos a continuación.

**Figura 4:**
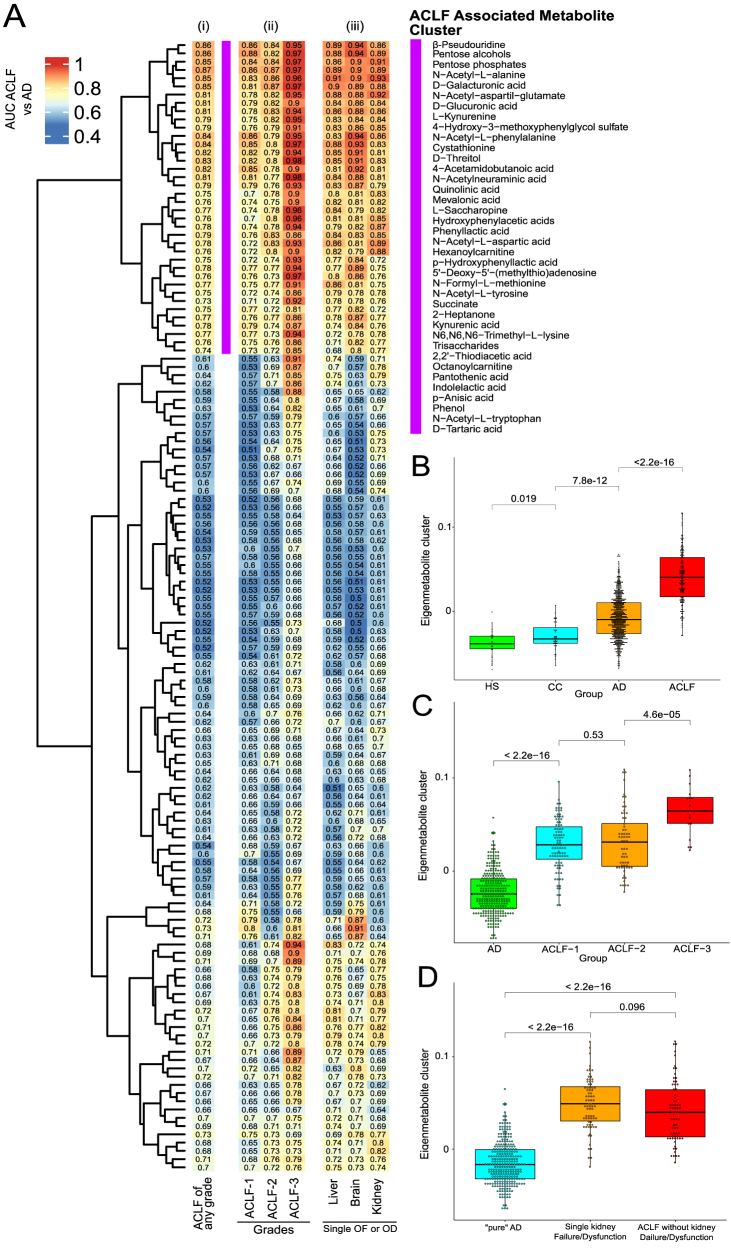
Huella metabólica específica de los pacientes con ACLF (A) y metabolito específico *(eigenmetabolite)* del grupo metabólico específico de la ACLF (B–D). Datos específicos de la referencia [49].

El aspecto más interesante del trabajo realizado por Moreau y col fue que identificaron un perfil plasmático compuesto de 38 metabolitos capaces de distinguir a los pacientes con ACLF de los que no la padecían [49] (Figura 4). La intensidad de esta huella metabolómica aumentaba conforme lo hacían los grados de ACLF y se mostró similar a la de los pacientes con insuficiencia renal y a la de aquellos que no la tenían, lo que indica que dicho perfil no solo reflejaba una excreción renal reducida, sino también un metabolismo celular alterado (Figura 4). Sin embargo, la relación directamente proporcional observada entre los niveles plasmáticos de marcadores inflamatorios (TNFα, CD206 soluble y CD163 soluble) y la intensidad de la alteración metabólica señala a la inflamación sistémica como su principal impulsora [49]. El objetivo de este intenso metabolismo catabólico en los pacientes con cirrosis AD es aportar los nutrientes necesarios para satisfacer la enorme cantidad de energía que la respuesta inflamatoria precisa, que debe ser suficiente para la producción de mediadores inflamatorios, la proliferación y migración de células inmunes, la explosión respiratoria y la producción de proteínas de fase aguda [59]. Esta respuesta inflamatoria sistémica tan demandante en términos de energía requiere la reasignación de la reserva de nutrientes para impulsar la activación del sistema inmune. Para tal fin, las células inmunes compiten por la energía con otros programas de mantenimiento, entre los que se encuentran aquellos responsables del funcionamiento adecuado de los órganos periféricos. Finalmente, esta compensación de energía entre la activación inmune y la homeostasis de los órganos puede provocar hipometabolismo periférico, disfunción e insuficiencia orgánica en estos pacientes.

El patrón plasmático específico de la ACLF también incluye metabolitos, lo que indica que el aumento de la glucólisis por los leucocitos circulantes es otro signo característico de la ACLF (Figura 4) [49]. En los mamíferos en condiciones normales, las células obtienen casi toda la energía de la fosforilación oxidativa mitocondrial (OXPHOS), que combina el transporte de electrones con la respiración celular y la síntesis de ATP [63]. Sin embargo, en condiciones inflamatorias, los mitocondrios se vuelven disfuncionales y las células pasan de producir ATP mediante OXPHOS a realizar glucólisis aeróbica [63]. La glucólisis aeróbica (también conocida como efecto Warburg) finalmente produce lactato, generando dos moléculas de ATP por cada molécula de glucosa, siendo así menos eficiente que la OSPHOS, que genera unas 36 ATP por cada molécula de glucosa [63]. Un aspecto importante a tener en cuenta es que el lactato, como producto de desecho de la glucólisis, es secretado en grandes cantidades por las células inmunes innatas al ser activadas, y este metabolito actúa como respuesta negativa para limitar la inflamación reduciendo la producción de citocinas y la migración de monocitos y macrófagos [64]. Sin embargo, una clara desventaja de la glucólisis es que esta depende en gran medida de la glucosa como única fuente de energía, mientras que la OXPHOS mitocondrial tiene más flexibilidad metabólica, pudiendo emplear, por ejemplo, ácidos grasos y aminoácidos como fuentes de carbono [63]. Los pacientes con cirrosis AD también presentan mayores niveles plasmáticos de productos intermedios de la vía pentosa fosfato, que se bifurca a partir de la glucólisis en el primer paso del metabolismo de la glucosa, lo que indica que el metabolismo de la glucosa citosólica a través de rutas alternativas a la glucólisis también es común en estos pacientes [59]. En las primeras fases, aún se mantiene la homeostasis de la energía desviando la producción de energía de la OXPHOS a la glucólisis. Sin embargo, esta es una solución a corto plazo, dado que las células no son capaces de mantener una producción elevada de energía mediante la glucólisis en fases avanzadas de la enfermedad hepática crónica [65].

El patrón metabólico específico de la ACLF también contiene metabolitos que indican una beta-oxidación mitocondrial de ácidos grasos productores de ATP. A este respecto, la inflamación sistémica promueve importantes cambios en la función mitocondrial de los órganos periféricos que impiden una adecuada generación de energía mitocondrial. De hecho, la inflamación sistémica inhibe tanto la translocación de los ácidos grasos a las mitocondrias como la beta-oxidación de los ácidos grasos, reduciendo la fosforilaciíon oxidativa y la producción de ATP [63]. En esta línea, en nuestro estudio, los niveles séricos de hexanoilcarnitina y tetradecenoilcarnitina, que son acilcarnitinas de cadena larga y media y son marcadores conocidos de beta-oxidación mitocondrial incompleta de ácidos grasos, estaban significativamente elevados en los pacientes con ACLF (Figura 4) [49].

## Panorama genómico y transcriptómico de la ACLF

La intensidad de la inflamación sistémica y la respuesta del sistema inmune a PAMP y DAMP en pacientes con cirrosis AD que desarrollan ACLF podría verse influida por factores genéticos del anfitrión. Así, se ha demostrado que algunos polimorfismos de nucleótido único (SNP) modulan la liberación de moléculas inflamatorias por parte de las células inmunes innatas o la inducción de cambios en la expresión de TLR. Por ejemplo, nuestro laboratorio analizó un panel de SNP localizados en seis genes diferentes fuertemente asociados con el proceso inflamatorio en una población de 279 pacientes con cirrosis AD, de los cuales 178 presentaban ACLF. Concretamente, analizamos dos SNP del clúster de genes IL-1 (rs1143623 SNP en el promotor del gen que codifica IL-1β, la citocina proinflamatoria clave que provoca la “tormenta de citocinas” y rs4251961, un SNP en el promotor del gen IL-1ra, que inhibe la respuesta inflamatoria antagonizando la unión de IL-1 a su receptor); un SNP (rs1800871) en el gen que codifica la citocina anti-inflamatoria IL-10 y otro SNP (rs4969170) en el gen supresor de la señalización de la citocina (SOCS) 3; el SNP rs31315500 que codifica la proteína que contiene el dominio de oligomerización de unión a nucleótidos (NOD2), un receptor que reconoce los lipopolisacáridos (LPS) bacterianos y se expresa principalmente en los leucocitos en sangre periférica; y finalmente, un SNP (rs1878022) en el gen receptor 1 de tipo quimiocina CMKLR1, que codifica un receptor acoplado a proteínas G que reconoce a RvE1, un pequeño mediador lipídico bioactivo con una potente actividad en la resolución de la inflamación (véase más arriba). Entre estos SNP, identificamos dos polimorfismos pertenecientes al clúster de genes IL-1 (IL-1α and ILα-1ra) El SNP IL-1α mostró ser un factor protector frente a la ACLF (OR: 0,34, IC95% 1,22–5,57, p<0,05) mientras que el SNP IL-1ra mostró una asociación dicotómica dependiente del modelo de herencia (superdominante: OR: 0,58, IC95% 0,35–0,95, p<0,05; co-dominante: OR: 2,61, IC95% 1,22–5,57, p<0,05). Cabe mencionar que se observó una menor frecuencia de ambas variantes en los pacientes con ACLF (20%) que en aquellos que no la padecían (80%). El papel funcional de la variante IL-1β quedó demostrado por una reducción en los niveles plasmáticos de IL-1β junto con la presencia de menores niveles circulantes de citocinas (p.ej. IL-1α, IL-6, la colonia de granulocitos que estimula el factor G-CSF y la colonia de granulocitos/macrófagos que estimula el factor GM-CSF), los niveles de PCR y el recuento de hematíes. La variante IL-1ra también resultó ser funcional y se asoció a cambios en los niveles de IL-ra y en los niveles circulantes de citocinas dependientes del genotipo [66]. En nuestro estudio identificamos dos polimorfismos funcionales comunes en el clúster de genes IL-1 con una fuerte asociación con el proceso inflamatorio relacionado con el desarrollo de ACLF. Más recientemente, se ha demostrado que las variantes genéticas en los genes que codifican los receptores del sistema inmunológico innato, como NOD2 o ligandos como la lectina de unión a manano (MBL) y las proteasas de serina asociadas a MBL (MAS) 2, están relacionadas con una mayor mortalidad a corto plazo en los pacientes con cirrosis AD y ACLF [67].

Aunque el análisis dirigido del genotipo ha sido instrumental a la hora de aportar la primera evidencia de la influencia del contexto genético en la evolución clínica de la enfermedad hepática avanzada, es esencial obtener una perspectiva más amplia de todo el genoma para lograr comprender de forma más completa la arquitectura genética de fenotipos complejos como el de la ACLF. De este modo, son necesarios más estudios de asociación de genoma completo para determinar el valor de las estrategias agnósticas en la identificación de variantes genéticas con implicaciones patofisiológicas. Tan y col. llevaron a cabo un estudio GWAS en 399 pacientes con ACLF relacionada con el virus de la hepatitis B (VHB) y 401 portadores asintomáticos del VHB sin tratamiento antiviral, e identificaron que el principal locus de susceptibilidad a ACLF relacionada con el virus de la hepatitis B es HLA-DR [68]. Actualmente se está llevando a cabo un proyecto de GWAS más ambicioso en más de 3000 pacientes con cirrosis AD y ACLF relacionada con el alcohol y/o con el virus de distintas zonas geográficas y diferente ascendencia. En este proyecto GWAS se está genotipando más de 75.000 SNP para obtener evidencia sólida sobre los determinantes genéticos en la patogénesis de la ACLF.

La transcriptómica ha evolucionado en la última década, habiéndose convertido en una herramienta esencial a la hora de investigar cambios en las condiciones inmunológicas, principalmente mediante la identificación de módulos de transcriptos con elevada coexpresión que identifiquen subconjuntos concretos de células inmunes [69]. Recientemente, Weiss y col realizaron un estudio transcriptómico de sangre pura en pacientes con ACLF caracterizada por una elevada leucocitosis, neutrofilia y linfopenia sanguínea. Estos autores observaron que tres módulos que contenían transcriptos relacionados con los neutrófilos y los monocitos estaban aumentados, mientras que cinco módulos que contenían transcriptos relacionados con la activación de las células dendríticas y los linfocitos, y con funciones efectoras y de memoria estaban deprimidos [70]. Estos hallazgos coinciden con la capacidad alterada de los pacientes con ACLF para desencadenar respuestas inmunes efectivas, por lo que se tienen un mayor riesgo de desarrollar infecciones graves y recurrentes. En los últimos tiempos, nuestro laboratorio ha aprovechado la transcriptómica para realizar un perfil de los efectos de la albúmina sérica humana sobre las células inmunes de pacientes con cirrosis AD y ACLF [71]. El hallazgo más relevante de este estudio es que la albúmina ejerce cambios importantes en el transcriptoma de las células inmunes, concretamente, en los genes relacionados con el compartimento endosómico implicado en la detección de ADN citosólico y en la respuesta del interferón tipo 1 [71]. También recientemente, Massey y col han realizado un análisis transcriptómico y metabolómico integrado, junto con una secuenciación de inmunoprecipitación acoplada a secuenciación de ADN, habiendo identificado al dominio hexoquinasa que contiene 1 como un nuevo biomarcador y diana terapéutica para pacientes con hepatitis y cirrosis alcohólica [72]. A través de la secuenciación de ARN, el mismo grupo también ha identificado que la expresión alterada del gen dependiente de HNF4a es un inductor de la insuficiencia hepática en pacientes con enfermedades hepáticas relacionadas con el alcohol [73]. Finalmente, la caracterización de miRNA en pacientes con cirrosis y ACLF han identificado un panel de 11 miRNA desregulados asociados a fallo orgánico, encefalopatía, infección bacteriana y un mal resultado clínico [74].

## Conclusiones

El síndrome de ACLF representa un nuevo paradigma entre las enfermedades caracterizadas por la presencia de una respuesta inflamatoria excesiva asociada al fallo orgánico o multiorgánico. De este modo, el síndrome de ACLF es una patología idónea para investigar los mecanismos subyacentes de la inflamación sistémica y el daño tisular, así como para identificar los factores desencadenantes de la respuesta inflamatoria sistémica asociada a la disfunción de órganos terminales. El síndrome de ACLF también ofrece la oportunidad de identificar nuevos biomarcadores, proceso que se está viendo acelerado por los actuales estudios que se están llevando a cabo mediante el uso de tecnologías de vanguardia y estudios ómicos integrados. La identificación y validación clínica de estos nuevos biomarcadores resultan de vital importancia para el desarrollo de nuevas terapias e intervenciones destinadas a reducir la inflamación sin inducir la inmunosupresión en estos pacientes.
